# Application of strain and other echocardiographic parameters in the evaluation of early and long-term clinical outcomes after cardiac surgery revascularization

**DOI:** 10.1186/s12872-019-1162-8

**Published:** 2019-08-05

**Authors:** Anna Gozdzik, Krzysztof Letachowicz, Barbara Barteczko Grajek, Tomasz Plonek, Marta Obremska, Marek Jasinski, Waldemar Gozdzik

**Affiliations:** 1 Department and Clinic of Cardiac Surgery, Wroclaw Medical University, Borowska 213, 50-556 Wroclaw, Poland; 2 Department and Clinic of Nephrology and Transplantation Medicine, Wroclaw Medical University, Wroclaw, Poland; 3 Department and Clinic of Anaesthesiology and Intensive Therapy, Wroclaw Medical University, Wroclaw, Poland; 4 Department of Medical Emergency, Wroclaw Medical University, Wroclaw, Poland

**Keywords:** Coronary surgery, Echocardiography, Longitudinal strain

## Abstract

**Background:**

Coronary artery bypass graft (CABG) surgery is an effective therapeutic strategy for coronary heart disease (CHD). Myocardial longitudinal strain echocardiography with 2D speckle tracking could obtain ventricular function with better accuracy and reliability than the left ventricular ejection fraction.

The aim of the study was to assess changes in left ventricular function in patients before and after surgical revascularization for a 24-month period of observation, using echocardiography with speckle tracking strain imaging. We searched for echocardiographic predictors of poor early and long-term outcome after CABG.

**Methods:**

We enrolled 69 patients scheduled for elective coronary bypass grafting. Patients were divided into groups based on pre-operative systolic and diastolic parameters, depending on the GLS value and the E’ Lat and E/E’ value. The correlation between these parameters and early and long-term outcomes was analyzed.

**Results:**

Preoperative EF was preserved in 86, 95% (60) patients. Pre-operative reduced GLS was observed in 73.91% (51) of patients and severely reduced in 31.88% (22).

In the first post-operative 6-month period, we observed a significant decrease in the GLS. The GLS was a predictor of early postoperative outcome for intubation time, the inotropes use and length of ICU stay. Diastolic dysfunction was a predictor of the greater inotrope requirements.

**Conclusions:**

Global longitudinal strain and diastolic dysfunction parameters are a good predictors of worse early outcome after CABG.

## Background

Coronary heart disease (CHD) is the leading cause of death worldwide. Coronary artery bypass graft (CABG) surgery is an effective therapeutic strategy for CHD which not only improves clinical symptoms but also reduces the risk of sudden cardiac death. Left ventricular (LV) systolic function is a powerful predictor of cardiovascular outcome and the severity of LV dysfunction is the main determinant in the decision to refer patients for cardiac surgery [1].

The most common echocardiographic measure of myocardial function is the left ventricle ejection fraction (LVEF). However, the accuracy and reproducibility of the LVEF measurement remains dependent on image quality, on ventricular loading conditions, heart rate and operator experience [2].

Assessing the myocardial global longitudinal strain (GLS) with 2D speckle tracking provides an accurate and reproducible measurement of regional and global LV contractility [3].

In recent studies that used magnetic resonance imaging (MRI), the strain was demonstrated to measure ventricular function with better accuracy and reliability than the left ventricular ejection fraction. Macron, et al. showed that in patients with poor acoustic windows, strain values correlated more closely with MRI measures of ventricular function than traditional EF [4].

Left ventricle diastolic dysfunction identified by echocardiography was strongly associated with reduced long-term freedom of major cardiac events (MACEs) in patients undergoing cardiac surgery revascularization. Swaminathan, et al. proposed a highly simplified algorithm using parameters E and E’ to classify the grade of the left ventricle diastolic dysfunction [5].

The aim of our study was to assess changes in LV function in patients before and after surgical revascularization using speckle tracking strain imaging over 24 months of observation.

We wanted to know if preoperative longitudinal strain indices and other selected indicators of LV diastolic function, especially in patients with preserved ejection fraction LV pEF (> 50%) could be independent and more sensitive predictors of early and long-term clinical outcomes after cardiac surgery.

## Methods

### Study population

Between February and August 2015, after review board approval and written informed consent, we enrolled 82 consecutive adult patients in the prospective single center clinical study. The patients were scheduled for first time elective coronary artery bypass grafting in the Cardiac Surgery Clinic of the Wroclaw Medical University, Poland.

Exclusion criteria were cardiac arrhythmias such as atrial fibrillation and paced rhythms, other valve operation and poor transthoracic echocardiography (TTE) imaging windows.

Preoperative demographic and clinical data like age, gender and the European System for Cardiac Operative Risk Evaluation Score (EuroSCORE II) were collected. Clinical data on any history of smoking, hyperlipidemia and statin therapy, hypertension and diabetes mellitus were also collected.

Surgical data included cardiopulmonary bypass (CPB) time and aortic cross clamp (ACC) time.

A two-year follow up was performed for 69 patients. Thirteen patients did not finish the follow-up period because of arrhythmia (4 pts), stroke (3 pts), and 5 patients did not appear during control visits. Three of them died after 2 years of observation for different causes (stroke and cancer). Three-year event-free survival was defined as the percentage of patients free from any major cardiac events or mortality from any cause up to 3 years after the date of study enrollment. It was ascertained by telephone follow up.

Echocardiographic examinations were performed before and at 3, 6, 12 and 24 months after surgery.

Postoperative outcome variables were duration of tracheal intubation (time from intensive care admission to extubation), length of intensive care unit (ICU) stay, length of hospital stay, inotrope requirements (dobutamine, milrinone, epinephrine [μg/kg/min]) and major cardiac events.

Major cardiac events per the Society of Thoracic Surgeon database were cardiac arrhythmias (ventricular tachycardia, ventricular fibrillation and asystole), multiple-organ failure, ventricular assist device (balloon pump or extracorporeal membrane oxygenation) and reoperation.

The EuroSCORE is used worldwide as a model for predicting mortality after cardiac surgery. The levels of risk mortality are divided into: Low (0–2.99%), Medium (3.0–5.99%) and High (> 6.0%).

In our study no one had a EuroSCORE above 2.99%.

EuroSCORE II includes: age, sex, renal impairment, presence/absence of chronic lung disease, extracardiac arteriopathy, poor mobility, previous cardiac surgery, active endocarditis, critical preoperative state, diabetes on insulin, unstable angina, recent myocardial infarction, pulmonary hypertension (moderate 30–55 mmHg, or severe > 55 mmHg) NYHA level, thoracic aortic surgery, number of valve procedures or isolated, urgency of intervention and LVEF (good > 50%, moderate 31–50%, poor 21–30% or very poor < 20%).

### Echocardiography

Comprehensive echocardiography was performed using the Vivid E9 ultrasound system (GE Vingmed, Milwaukee, WI, USA) equipped with an M3 S transducer.

Echocardiograms were performed in accordance with the guidelines of the American Society of Echocardiography (ASE) and the European Association of Echocardiography (EAE) for the evaluation of cardiac chamber size and valve function. The left ventricular ejection fraction (LVEF) was measured using the Simpson biplane method of disks from apical four and two chamber views. The left ventricular mass index (LVMI) and relative wall thickness (RWT) were calculated according to ASE/EAE guidelines. The peak velocities of the E wave (early diastole) and the A wave (late diastole) were measured by pulsed-wave (PW) Doppler at the level of the mitral valve tips and the ratio of these velocities was calculated. Pulsed-wave Tissue Doppler was used, and peak velocities were obtained at the level of the lateral and septal mitral annulus during early diastole E’ and late diastole A’ and systole S′. The E/E’ ratio was then calculated. Using the simplified algorithm proposed by Swaminathan, patients were classified into 3 grades of left ventricular diastolic dysfunction. Tricuspid annular plane systolic excursion (TAPSE) was also measured.

### 2D speckle tracking echocardiography

Apical four, two and three chamber views were used (4Ch, 2CH, LAX) to calculate the LV GLS average. Left ventricular strain quantification was then performed offline using commercially available software (Echo PAC; GE Healthcare). To calculate the LV GLS, a line was traced along the endocardium’s inner border in each of the three apical views on an end-systolic frame and a region of interest was automatically defined between the endocardial and epicardial borders.

Left ventricular global longitudinal strain was obtained by averaging all segmental 3 apical view strain values. Peak global strain was defined as the peak negative value on the strain curve during the entire cardiac cycle. Due to the fact that LV GLS values are negative, a lower absolute number represented a worse value than a higher one.

In patients with coronary artery disease a longitudinal strain cut off of − 14.1% is used to detect ischemic myocardial segments. The cut off of − 18.6% GLS is the normal value [6].

Patients were divided into groups according to preoperative measurements of systolic and diastolic function.

Depending on the LVEF value:

- EF < 50% moderately impaired EF
- EF ≥50% normal EF

Depending on the GLS average value:

- Group 1: GLS avg. > − 18.6% normal longitudinal strain.
- Group 2: GLS avg. between − 14.0% and − 18.6% moderately reduced longitudinal strain.
- Group3: GLS avg. < − 14.0% heavy reduced longitudinal strain.

Depending on the value of E/E’ and E’:

- E/E’ > =12
- E’ < 10 cm/s

### Statistical analysis

The results were collected and systematized using Excel 2016. The statistical analysis was performed using Statistica 13.1 PL. At the beginning, basic descriptive statistics of the variables examined were calculated. Due to the rejection the hypothesis and normality of the data distribution by the Shapiro-Wilk test in further analysis the U Mann Whitney test and the Friedman ANOVA (with the post hoc test) were used respectively.

Relationships between examined parameters were searched using Spearman’s rank order correlation. Distributions of qualitative variables were examined using cross-reference tables with the Pearson chi square test, chi square of the highest credibility, or chi square with the Yates correction as appropriate.

The area below the receiver operating characteristic (ROC) curve was used to determine the sensitivity and specificity of the continuous variables that displayed statistical significance.

## Results

### Patient characteristics

Finally, 69 patients (55 male, 79.71%), 65 (13) years old undergoing elective coronary artery bypass grafting were included in the two-year period of observation time.

Mean cardiopulmonary bypass time was 82 (34) min with an aortic cross clamp time of 42 (21) min.

Fifty-five patients had 3-vessel coronary artery disease, including significant left main stenosis in 24 patients, and seven patients had 2 vessel disease, two with significant left main stenosis. The majority of patients received three bypass grafts (median 3, range 2–4) and in all except for 7, a left mammary artery to the left anterior descending coronary artery bypass graft was used.

Baseline characteristics and operative data are shown in Table 1.

**Table 1:** Baseline characteristics and operative data in all and GLS groups

	All patients *N*= 69	Group 1 (*n* =18) GLS 1 < -18,6	Group 2 (*n*=29) -18,6 ≤ GLS 1 ≤ -14	Group 3 (*n*=22) GLS 1 > -14
	Mean (SD)	Mean (SD)	Mean (SD)	Mean (SD)
Age, years	65.0 (8.7)	63.89 (11.1)	65.79 (8.0)	65.18 (7.9)
BMI, kg/m2	27.9 (4.0)	25.3 (3.5)	28.5 (2.9)	29.6 (3.1)
Gender M, (%)	55 (79.7%)	14 (77.7%)	21 (72.4%)	20 (90.9%)
Hypertension, (%)	54 (78.2%)	14 (77.7%)	22 (75.8%)	18 (81.80%)
Gender M, (%)	55 (79.7%)	14 (77.7%)	21 (72.4%)	20 (90.9%)
Hypertension, (%)	54 (78.2%)	14 (77.7%)	22 (75.8%)	18 (81.80%)
Diabetes, (%)	25 (36%)	5 (27.7%)	10 (34.4%)	10 (45.4%)
EURO Score	0.007 (0.002)	0.007 (0.002)	0.007 (0.002)	0.008 (0.002)
LM stenosis, (%)	25.71	25.5	23.10	29.27
CPB time, (min.)	75.9 (34.6)	73.8 (31.3)	78.9 (43.2)	73.6 (32.3)
Aortic cross clamp time, (min.)	41 (21.7)	41,2 (20.5)	43.8 (25,9)	37.2 (14.3)

*Abbreviations*: *BMI* body mass index, *CPG* cardiopulmonary bypass, *GLS* global left ventricular longitudinal strain, *LM* left main stenosis

### Preoperative parameters

Preoperative Ejection Fraction (EF) was averaged 58.04% (range, 30–73%). LVEF was preserved (≥ 50%) in 60 patients (86.95%) and moderately impaired EF < 50% (30–50%) in 9 patients (13.05%).

Based on the pre-operative values of GLS we separated patients into 3 groups.

- Group 1 - GLS > -18.6%. (*n* = 18) normal GLS.
- Group 2 - GLS between − 14.0% and − 18.6% (*n* = 29) moderately reduced GLS.
- Group 3 – GLS ≤ -14.0% (*n* = 22) severely reduced GLS.

A preoperative reduced GLS was observed in 73.91% of patients and severely reduced in 31.88%. Among patients with a preserved LVEF (*n* = 60), severely impaired GLS (<− 14%) was observed in 12 patients (20%). Comparison of global longitudinal strain in patients with/ or without left main stenosis (LMS) (− 15.86% vs. -16.76% *p* = 0.39), and with/or without LAD stenosis (− 16.21 vs. -18.12% *p* = 0.32), and with /or without CX stenosis (− 15.86 vs. -16.91 *p* = 0.35) did not revealed any significant differences. There was no correlation between left ventricular hypertrophy and GLS, although there was a weak correlation with left ventricular mass (LVM) (correlation ratio = 0.257 *p* < 0.05).

The men had the lowest values of GLS before the operation than the women (− 16.07% vs. -17.45%), however, without statistical significance *p* = 0.27.

Left ventricular diastolic dysfunction (LVDD) was observed in 39 of patients with E’ Lat’ < 0.10 (56.5% of the whole group). Ten patients (14.4%) had E/E’ > 12.

Diastolic function parameters are shown in Table 2.

**Table 2:** Postoperative outcome in patients with preoperative left ventricular diastolic dysfunction with E’ < 10 or E/E’ >12

Variables	All pts *n* = 69	E’ < 0,10 m/s *n*=39	E’ ≥ 0,10 m/s *n*=30	p	E/E’ >12 *n*=10	E/E’≤12 *n*=59	P
Intubation time, h/min	5.46	7.17	3.48	0.32	14.51	4.12	0.04^a^
ICU time, days	2.66	2.76	2.53	0.31	2.70	2.66	0.71
Hospitalization time, days	9.94	10.07	9.76	0.33	9.70	9.98	0.72
Inotrops Use, %	28.98	78.95	13.79	0.04^a^	60	22.4	0.02^a^
MCE, %	24.63	23.08	26.67	0.71	30	23.7	0.61
1-year event free, %	12.06	8.33	13.79	0.44	20	9.8	0.32
2018 Phone call, %	8.06	8.11	6.67	0.82	20	5.2	0.11

*Abbreviations*: *E’* early diastolic mitral annulus velocity, *E* early transmitral inflow wave velocity, *ICU* intensive care unit, *MCE* major cardiac events

^a^Statistically significant

### Postoperative outcome

The ejection fraction measured by 2D echocardiography in the group with preserved LVEF (*n* = 60) decreased slightly after surgery from EF 61% preoperatively to EF 59.8%, while in patients with moderately impaired LVEF (*n* = 9) there was an increase from EF 31.3% to EF 41.6% after 12 months. It was not statistically significant.

Myocardial mechanics measured by speckle tracking echocardiography (GLS) in the whole group (*n* = 69) revealed a decrease in measurements taken on the day of discharge from the hospital and one and 3 months after discharge. The mean values were significantly lower relative to the results gained before surgery, respectively − 16.7% vs. -14.7% *p* = 0.01; − 16.1% vs. -14.5% *p* = 0.004; 15.9% vs. -14.5% *p* = 0.03.

After 12 and 24 months the global longitudinal strain was slightly increasing however, without statistical significance (− 16.3% vs. -15.3% *p* = 0.06 and − 16.1% vs. -15.7% *p* = 0.06).

A similar observation of a reduction in GLS values during the first 6 postoperative months was noted independently in two groups, separated based on preoperative GLS values.

In Group 1 with GLS > -18.6% the values slightly increased until after 12 and 24 months from surgery (GLS − 21.6% before to − 18.4% and − 18.6%, respectively) and in Group 2 with GLS − 14% to − 18.6% (from − 16.1% to − 15.7% and − 15.6%, respectively).

In Group 3 with GLS ≤ -14% the observations were different. At the beginning of the postoperative examinations there was an increase in the GLS, but it was still low: from a value of − 11.6% preoperatively to − 12.8% at discharge, to − 13.4% after 6 months, − 12.8% after 12 months and − 13.3% after 24 months. It was not statistically significant.

In searching for predictors of early and long-term clinical outcomes we found the following results.

Patients with an impaired LVEF < 50% (*n* = 9) had prolonged intubation time (16 h00’ vs. 4 h14’ *p* = 0.03) and greater inotrope requirements (66.67% vs. 22.03% *p* = 0.01) compared to those with preserved EF ≥50% (*n* = 60).

In the group with severely reduced GLS > -14% intubation time was longer (9 h02’ vs. 3 h26’ vs. 4 h21’ *p* = 0.37) and inotrope requirements were greater (41.67% patients vs. 25 and 12.5%, respectively *p* = 0.10) in relation to Groups 2 and 3, but it was not statistically significant.

When we looked at the GLS of the whole group of examined patients we found significant correlation between preoperative GLS and intubation time (*p* = 0.01) and inotrope use (*p* = 0.03). Based on the ROC curve analysis, GLS seems to be a good predictor of early postoperative outcome with the: prolongation of intubation time (AUC 0,634 *p* = 0,049), greater requirements for inotrope use (AUC 0,653 *p* = 0,038) and longer intensive care unit stay time (AUC 0,634 *p* = 0,049).

The hospitalization and intensive care unit stay time did not differ significantly in those groups. The 3-year cardiac-event free survival and MCE were also similar.

In the group of patients with E/E’ > 12 (*n* = 10) in the postoperative period we observed a statistically significant prolonged intubation time (14 h51’ vs. 4 h14’ *p* = 0.04) and greater inotrope requirements (60.0% vs. 22.41% *p* = 0.02) compared to patients with E/E’ ≤ 12. The ROC analysis showed that E/E’ is a good predictor only of early outcome: greater inotropes requirements (AUC 0,764 and *p* = 0,0001).

There were no differences in intensive care unit and hospitalization stay time and MCE and 3-year cardiac-event free survival.

The patients from the group with Lat E’ < 0.10 (*n* = 39) had significantly greater inotrope requirements (38.46% vs. 13.79% *p* = 0.04) than patients with Lat E’ > 0.10. Parameter E’ in ROC analysis was not a good predictor of early and long outcome.

The group of patients with an elevated EuroSCORE II (*n* = 9) over 1% had: intubation time (16 h33’ vs. 4 h9’ *p* = 0.00), MCE (55.56% vs. 20.0% *p* = 0.03) inotrope requirements (44.44% vs. 25.42% *p* = 0.00), one-year event-free survival (37.50% vs. 7.02% *p* = 0.03) significantly worse than the patients with a EuroSCORE II < 1%.

Relative wall thickness (RWT) was not a predictor of worse outcome.

Echocardiographic indices during the observation time are shown in Tables 3, 4, 5, 6.

**Table 3:** Pre and post-operative measurements in whole observation group (*n*=69)

Variables	Preoperative	6 months	12 months	24 months	
	Mean (SD)	Mean (SD)	Mean (SD)	Mean (SD)	p
LVDD, mm	48.9 (5.9)	49.6 (5.5)	48,9 (5.6)	49.8 (5.6)	0.81
E/E'	9.0 (4.2)	8.0 (4.5)	7.8 (3.5)	7.8 (4.3)	0.90
E’, m/s	0.09 (0.03)	0.11 (0.03)	0.11 (0.03)	0.11 (0.03)	0.01^a^
EF, %	58.0 (9.5)	57.5 (8.2)	57.4 (9.4)	55.9 (9.7)	0.70
GLS, %	-16.1 (4.2)	-14.4 (3.2)	-15.5 (4.1)	-15.7 (3.9)	0.41
TAPSE, mm	23.0 (3.7)	18.7 (2,8)	19.7 (2.2)	20.7 (1.8)	0.00^a^

*Abbreviations*: *E* early diastolic transmitral inflow wave velocity, *E’* early diastolic mitral annulus velocity, *EF* ejection fraction, *GLS* global left ventricular longitudinal strain, *LVDD* left ventricular diastolic diameter, *TAPSE* tricuspidal annular plane systolic excursion

^a^Statistically significant

**Table 4:** Pre and post-operative measurements in group 1

Variables	Group 1 = GLS Av1< -18,6				
	Pre-operative	6 months	12 months	24 months	p
	Mean (SD)	Mean (SD)	Mean (SD)	Mean (SD)	
LVDD, mm	47,1 (4,7)	46 (4,5)	47,5 (5,3)	47,1 (4,8)	0,40
E/E'	7,9 (4,1)	9,8 (9,4)	6,6 (2,6)	6,4 (2,9)	0,91
E’, m/s	0,11 (0,03)	0,12 (0,05)	0,13 (0,03)	0,13 (0,03)	0,11
EF, %	60,0 (7,0)	60,0 (7,0)	65,0 (-)	60,0 (7,0)	0,42
GLS, %	-20,7 (0,7)	-15,7 (0,5)	-16,6 (0,7)	-17,5 (0,7)	0,21
TAPSE, mm	22,5 (3,5)	17,0 (4,2)	19,0 (2,8)	18,5 (0,5)	0,20

*Abbreviations*: *E* early diastolic transmitral inflow velocity, *E’* early diastolic mitral annulus velocity, *EF* ejection fraction, *GLS* global longitudinal left ventricular strain, *LVDD* left ventricular diastolic diameter, *TAPSE* tricuspidal annular plane systolic excursion

**Table 5:** Pre and post-operative measurements in group 2

Variables	Group 2 = -18,6 ≤ GLS 1 ≤ - 14				
	Preoperative	6 months	12 months	24 months	
	Mean (SD)	Mean (SD)	Mean (SD)	Mean (SD)	p
LVDD, mm	48,2 (5,7)	48,9 (4,9)	48,2 (5,6)	49,5 (5,6)	0,30
E/E'	8,3 (3,2)	7,1 (2,1)	7,7 (4,7)	8,1 (4,7)	0,92
E’, m/s	0,07 (0,2)	0,08 (0,02)	0,10 (0,00)	0,11 (0,02)	0,51
EF, %	63,3 (2,8)	61,6 (5,7)	61,6 (5,7)	61,3 (6,30	0,40
GLS, %	-16,2 (1,3)	-14,6 (2,3)	-15,7 (3,56)	-15,6 (3,2)	0,62
TAPSE, mm	24,6 (6,6)	18,0 (2,6)	17,0 (1,0)	20,3 (0,5)	0,32

*Abbreviations*: *E* early diastolic transmitral inflow wave velocity, *E’* early diastolic mitral annulus velocity, *EF* ejection fraction, *GLS* global longitudinal left ventricular strain, *LVDD* left ventricular diastolic diameter, *TAPSE* tricuspidal annular plane systolic excursion

**Table 6:** Pre and postoperative measurements in group 3

Variables	GLSAv1 Group 3=GLSAv1>-14				
	Pre-operative	6 months	12 months	24 months	
	Mean (SD)	Mean (SD)	Mean (SD)	Mean (SD)	p
LVDD, mm	51,5 (6,5)	53 (5,5)	51,1 (6,1)	52,4 (5,9)	0,60
E/E'	9,0 (3,9)	7,0 (3,0)	6,6 (3,0)	6,6 (2,7)	0,83
E', m/s	0,07 (0,04)	0,11 (0,02)	0,12 (0,06)	0,14 (0,02)	0,06
EF, %	46,6 (10,4)	50,0 (10,0)	50,0 (10,0)	50,0 (10,0)	0,21
GLS, %	-11,6 (1,9)	-13,4 (4,3)	-12,8 (3,9)	-13,3 (3,6)	-
TAPSE, mm	22,2 (1,6)	19,8 (2,6)	21,6 (0,8)	21,8 (1,9)	0,01^a^

*Abbreviations*: *E* early diastolic transmitral inflow wave velocity, *E’* early diastolic mitral annulus velocity, *EF* ejection fraction, *GLS* global longitudinal left ventricular strain, *LVDD* left ventricular diastolic diameter, *TAPSE* tricuspidal annular plane systolic excursion

^a^Statistically significant

## Discussion

The aim of our study was to assess changes in the LV function before and after surgical cardiac revascularization using 2D transthoracic echocardiography with speckle tracking imaging in a 24-month observation period, attempting to find echocardiographic parameters that would predict early and long-term clinical outcomes in patients after CABG surgery.

Our study showed that GLS was reduced before revascularization in the majority of patients, despite a preserved left ventricular ejection fraction.

This parameter significantly decreased during the first 3 months after surgery, whereas the left ventricular diastolic dysfunction parameters observed in this group of patients before surgery improved after CABG.

GLS and diastolic parameters expressed by E/E’ were predictors of early outcome after CABG.

The left ventricular systolic function is primarily assessed using the LV ejection fraction (LVEF) based on 2D echocardiographic imaging. EuroSCORE II includes LVEF for the stratification of operative risk in patients scheduled for cardiac surgery [7, 8]. The accuracy and reproducibility of the LVEF measurement depends on the image quality, operator experience, dependence on the ventricular load, and heart rate and only assesses the global function without regional myocardial abnormalities.

Assessment of the longitudinal function of the myocardium by means of speckle tracking has been proposed to overcome these problems. Speckle tracking can assess the global and regional function of the myocardium and provide reproducible data on myocardial deformation [9].

Macron et al. demonstrated that GLS assessed by 2D speckle tracking is better correlated with LVEF assessed by MRI than LVEF assessed by means of echocardiography [10]. Also, in the study of Brown, et al. it was noted that in patients with more than five abnormal contractile segments, GLS is better correlated with LVEF by MRI than LVEF derived from 2D echocardiography [11].

GLS should not be assimilated to LVEF, because longitudinal myocardial deformation is mainly driven by the subendocardial layers. The impaired myocardial contractility in ischemic heart disease affects the subendocardial layers – the longitudinal fibers first. The fibers from mid-myocardial and subepicardial layers are responsible for radial and circumferential deformation and are less sensitive, and the impairment of their function appears later. They have less sensitivity in detecting pathology. The LVEF provides information about the function of the mid-myocardial layer – the radial fibers. GLS not only makes it possible to obtain a quantitative assessment of myocardial function but also allows for changes in myocardial interstitium, including the extent of myocardial fibrosis. This may explain the incremental value of GLS over LVEF for detecting early changes in myocardial function and for predicting cardiovascular outcome [12, 13].

We have observed that the values of myocardial strain after surgery depend on the pre-operative values. During the first 6 months after surgery, a reduction in global longitudinal strain was observed in patients with normal value or moderate preoperative impairment. Patients with severe preoperative impairment of global longitudinal strain had a slight improvement in postoperative parameters.

This is irrefutable evidence that speckle tracking echocardiography can detect subclinical left ventricular dysfunction and should be the primary tool for pre- and post-operative evaluation of the left ventricular systolic function in patients scheduled for CABG [14].

This opinion was also confirmed by Duncan, et al. in their review which describes the clinical use of myocardial deformation and its usefulness in anesthesia practice.

A deterioration of the LV systolic function after CABG surgery can take place in three different ways: 1) as a result of intraoperative global ischemia, 2) myocardial stunning or 3) early post-operative graft failure [15].

In the available literature there are not many reports describing the use of GLS to assess changes in LV systolic function after CABG, especially in patients with LVpEF. It is mainly due to a lack of recommendations for its use in routine echocardiography after CABG.

From a study by Koene R.J., et al. we know that patients after CABG with preserved pre-operative EF had a statistically significant decrease in LVEF [16]. Patients with preoperative LV dysfunction had a significant improvement in LVEF in a follow-up time ranging from 3 to 24 months. The author explained this observation by the relationship between factors associated with CABG and stated the need for prospective studies confirming this observation [17, 18]. Several studies have shown an improvement in LV function after CABG in patients with reduced LV function [19–21].

An explanation of this phenomenon may be the fact that myocardial improvement in the ischemic regions requires considerable time after revascularization and it depends on several factors. In addition, cardiac arrest, extracorporeal circulation and other surgical procedures when CABG surgery is performed may also worsen ischemic reperfusion injury, especially to the subendocardial muscle fibers. As a consequence of surgical trauma, there may be edema and a contraction of the ventricular wall and hemorrhagic cell infiltration [22]. This process may delay the recovery of the myocardial function.

The left ventricular systolic function is a strong predictor of cardiac surgery outcomes.

A reduction in preoperative ventricular systolic function can lead to hemodynamic instability in the postoperative period, requiring inotropic support, a prolonged hospital stay and can result in long-term complications, including increased mortality.

In searching for predictors of early and long-term clinical outcomes we found that patients with severely impaired GLS < -14% tended to have elongated intubation time and greater inotrope requirements (41.67% patients vs. 25 and 12.5%, respectively), although there were no statistically significant differences (*p* = 0.37; *p* = 0.11). The entire group observations indicated that GLS was a good predictor of early outcome relative to prolonged intubation time, greater inotrope use and prolonged ICU stay.

Ternacle, et al. in their study on 425 patients, indicated that GLS has incremental value for predicting the combined endpoint defined by death and the need for prolonged inotropic support. Within this group, survival occurred when the GLS was − 14 ± 4% and death when − 12 ± 4% *p* = 0.006 and inotropic support when GLS − 12 ± 4% and no inotropic support with GLS − 15 ± 4% *p* < 0.001.

Left ventricular diastolic dysfunction (LVDD) is a useful marker of mortality and morbidity in cardiac surgery patients. Afilalo J, et al. concluded in their study that preoperative echocardiography with restrictive left ventricular filling, which means 3 degrees of LVDD, provides incremental prognostic value in identifying patients at higher risk of mortality or major morbidity after coronary artery bypass surgery.

We observed in our study 39 patients with E’ < 10 cm/s. They all had significantly worse early outcome with greater inotrope requirements (38.46% vs. 13.79% *p* = 0.048) than patients without LVDD. In the group of patients with E/E’ > 12 (*n* = 10) we observed statistically significant prolongation of intubation time and greater inotrope requirements. After ROC analysis we can indicate that LVDD is a good predictor of early postoperative outcome of greater inotrope requirements. Gjertsson, et al. in their study found that moderate to severe diastolic dysfunction implies nonreversible myocardial changes that negatively affect survival [23].

Also, Lee EH, et al. showed that the E/e’ ratio is an independent predictor of 30-day and 1-yr MACE in patients who undergo elective off-pump coronary artery bypass graft surgery [24].

Left ventricular systolic function is routinely assessed by the left ventricular ejection fraction (EF). It is widely known that a preoperative decrease in ventricular function can lead to a poor postoperative course.

In our study, patients with an impaired LVEF < 50% (*n* = 9) had prolonged intubation time (16 h00’ vs. 4 h14’ *p* = 0.031) and greater inotrope requirements (66.67% vs. 22.03% *p* = 0.011) compared to those with a preserved EF ≥50% (*n* = 60).

We are aware that our study has a number of limitations. This is mainly due to the size of the studied group of patients. Irrespective of this, our study revealed certain trends and confirmed the usefulness of the described echocardiographic methods for assessing left ventricular systolic and diastolic function in patients undergoing CABG.

Relatively few patients did not come to the control examination. One explanation may be that the patients were mostly asymptomatic after the CABG surgery and in their opinion, they saw no reason to submit to a follow-up examination.

## Conclusions

Global longitudinal strain assessed by 2D speckle tracking is a highly sensitive tool to obtain left ventricular systolic mechanics in patients scheduled for coronary bypass grafting, especially with a preserved left ventricular systolic function (LVpEF).

Our study results showed that GLS was a good predictor of early postoperative outcome.

Left ventricular diastolic evaluation by the E and E’ parameters could predict early outcome after surgery coronary revascularization.

## Data Availability

The datasets analyzed during the current study are available from the corresponding author on reasonable request.

## Abbreviations

2D: Two dimensional
4CH,2CH,LAX: Four, two chamber views, long axis view
ACC: Aortic cross clamp
ASE: American Society of Echocardiography
CABG: Coronary artery bypass grafting
CHD: Coronary heart disease
CPB: Cardiopulmonary bypass
CX: Circumflex artery
E: Early mitral diastolic inflow
E’: Early tissue doppler diastole
EAE: European association of echocardiography
EuroSCOREII: European system for cardiac operative risk evaluation score
GLS: Global longitudinal strain
ICU: Intensive care unit
LAD: Left anterior descending artery
LVDD: Left ventricular diastolic dysfunction
LVEF: Left ventricular ejection fraction
LVMI: Left ventricular mass index
MACEs: Major cardiac events
MRI: Magnetic resonance imaging
PW: Pulse wave
RWT: Relative wall thickness
TAPSE: Tricuspid annular plane systolic excursion
TTE: Transthoracic echocardiography
